# Supplementary material for: The SARS-CoV-2 nucleocapsid protein is dynamic, disordered, and phase separates with RNA
Source: Nat Commun. 2021 Mar 29;12:1936. doi: 10.1038/s41467-021-21953-3 (PMC8007728; doi:10.1038/s41467-021-21953-3)
Supplement: Supplementary file 3 — Reporting Summary [file 41467_2021_21953_MOESM3_ESM.pdf]

nature research

Corresponding author(s): Alex S. Holehouse & Andrea Soranno

Last updated by author(s): 17th Feb 2021

Reporting Summary

Nature Research wishes to improve the reproducibility of the work that we publish. This form provides structure for consistency and transparency in reporting. For further information on Nature Research policies, see our Editorial Policies and the Editorial Policy Checklist.

Statistics

For all statistical analyses, confirm that the following items are present in the figure legend, table legend, main text, or Methods section.

n/a	Confirmed	
☐	☒	The exact sample size (*n*) for each experimental group/condition, given as a discrete number and unit of measurement
☐	☒	A statement on whether measurements were taken from distinct samples or whether the same sample was measured repeatedly
☒	☐	The statistical test(s) used AND whether they are one- or two-sided<br>*Only common tests should be described solely by name; describe more complex techniques in the Methods section.*
☒	☐	A description of all covariates tested
☒	☐	A description of any assumptions or corrections, such as tests of normality and adjustment for multiple comparisons
☐	☒	A full description of the statistical parameters including central tendency (e.g. means) or other basic estimates (e.g. regression coefficient) AND variation (e.g. standard deviation) or associated estimates of uncertainty (e.g. confidence intervals)
☒	☐	For null hypothesis testing, the test statistic (e.g. *F*, *t*, *r*) with confidence intervals, effect sizes, degrees of freedom and *P* value noted<br>*Give P values as exact values whenever suitable.*
☒	☐	For Bayesian analysis, information on the choice of priors and Markov chain Monte Carlo settings
☒	☐	For hierarchical and complex designs, identification of the appropriate level for tests and full reporting of outcomes
☒	☐	Estimates of effect sizes (e.g. Cohen's *d*, Pearson's *r*), indicating how they were calculated

Our web collection on statistics for biologists contains articles on many of the points above.

Software and code

Policy information about availability of computer code

Data collection	All software used to perform data collection is provided as open-source packages as detailed in the methods sections. CAMPARI V2 (http://campari.sourceforge.net/) was used for all-atom simulations using the ABSINTH 3.2 OPLS/AA forcefield. PIMMS (Python 3 implementation V0.1.27) was used for coarse-grained simulations. GROMACS 2019 was used for local simulations, whereas the Folding@Home platform uses GROMACS 5., n both cases with the AMBER03 force field with explicit TIP3P solvent. Additional input files for simulation are available from https://github.com/holehouse-lab/supportingdata/tree/master/2021/cubuk_nucleocapsid_2021.
Data analysis	All analysis was performed using open-source tools as detailed in the methods section. CAMPARITraj v0.1.2 was used for the analysis of all-atom simulations (https://github.com/holehouse-lab/camparitraj). Any specific analysis code that builds on publicly available software tools is available upon request from the authors. Data supporting the findings of this manuscript are available from the corresponding authors upon request. Simulation parameters used (including keyfiles for all-atom Monte Carlo simulations) are available from https://github.com/holehouse-lab/supportingdata/tree/master/2021/cubuk_nucleocapsid_2021.

For manuscripts utilizing custom algorithms or software that are central to the research but not yet described in published literature, software must be made available to editors and reviewers. We strongly encourage code deposition in a community repository (e.g. GitHub). See the Nature Research guidelines for submitting code & software for further information.

Data

Policy information about availability of data

All manuscripts must include a data availability statement. This statement should provide the following information, where applicable:

- Accession codes, unique identifiers, or web links for publicly available datasets
- A list of figures that have associated raw data
- A description of any restrictions on data availability

All data (raw and processed) are available upon request from the authors.

Field-specific reporting

Please select the one below that is the best fit for your research. If you are not sure, read the appropriate sections before making your selection.

☒ Life sciences ☐ Behavioural & social sciences ☐ Ecological, evolutionary & environmental sciences

For a reference copy of the document with all sections, see nature.com/documents/nr-reporting-summary-flat.pdf

Life sciences study design

All studies must disclose on these points even when the disclosure is negative.

Sample size	The sample size for all free diffusion measurements was always > 750 bursts.
Data exclusions	For all single-molecule experiments, exclusion of bursts from single-molecule data has been decided based on the following quantitative criteria. Based on the histogram of photon counts of the overall measurements (with 1 ms binning), a selection threshold for burst acceptance has been defined extrapolating the boundaries from the background contribution. We further rejected molecules that exhibit a much greater number of photons (>300) than the average distribution of molecules and that represents aggregated species of the protein or buffer impurities. Finally, based on Pulsed-Interleaved-Excitation (PIE) we excluded bursts that correspond to molecules with labeling stoichiometry corresponding to donor and acceptor only molecules, which therefore do not contribute to FRET. For all-atom simulations of RBD-Linker-DIM three of the 31 independent trajectories became kinetically trapped, an issue that, given the size of the system and the nature of Monte Carlo simulations is not unexpected. These three simulations were excluded from ensemble average calculations to avoid a relatively large contribution for a small number of states. Exclusion criteria were defined prior to simulations for this specific system based on the possibility of long-lived artificially kinetically-metastable states for such a larger system.
Replication	For single-molecule experiments: The sample size for all free diffusion measurements was always > 750 bursts after exclusion of acceptor- and donor- only molecules, corresponding to at least 750 double-labeled independent molecules in each measurement. For all-atom simulations of IDRs in isolation thirty independent simulation replicas were run. For all-atom simulations of IDRs in the context of folded domains, when Folding@Home simulations were run to generate starting configurations 200 independent simulations were run. When a single starting structure was used (i.e. in simulations of RBD-Linker-DIM) 31 independent simulations were run. The number selected simulations run is ~10x larger than the commonly used 3 independent replicas in all-atom simulations to ensure good convergence in our ensemble. For coarse-grained simulations, at least 3 independent replicas were run per condition.
Randomization	No randomization was used, as randomization was not appropriate at any stage in the study.
Blinding	Experiments were not blinded, as blinding was not appropriate at any stage in the study.

Reporting for specific materials, systems and methods

We require information from authors about some types of materials, experimental systems and methods used in many studies. Here, indicate whether each material, system or method listed is relevant to your study. If you are not sure if a list item applies to your research, read the appropriate section before selecting a response.

Materials & experimental systems

n/a	Involved in the study	
☒	☐	Antibodies
☒	☐	Eukaryotic cell lines
☒	☐	Palaeontology and archaeology
☒	☐	Animals and other organisms
☒	☐	Human research participants
☒	☐	Clinical data
☒	☐	Dual use research of concern

Methods

n/a	Involved in the study	
☒	☐	ChIP-seq
☒	☐	Flow cytometry
☒	☐	MRI-based neuroimaging
